# Supplementary material for: Cell cycle dependent coordination of surface layer biogenesis in Caulobacter crescentus
Source: Nat Commun. 2024 Apr 18;15:3355. doi: 10.1038/s41467-024-47529-5 (PMC11026435; doi:10.1038/s41467-024-47529-5)
Supplement: Supplementary file 1 — Supplementary Information [file 41467_2024_47529_MOESM1_ESM.pdf]

SUPPLEMENTARY INFORMATION

Supplementary Table 1. Strains and plasmids used in this study.

Strain	Description	Source	Citation
E. coli LMG194	Expression strain for pBAD plasmids	ATCC 47090	
E. coli BL21 (DE3)	Expression strain for pDEST14 plasmids	ThermoFisher Cat # EC0114	
C. crescentus CB15N (NA1000)	Wildtype *C. crescentus* strain	ATCC 19089	
C. crescentus CB15N Δ*sapA rsaA467:SpyTag*	RsaA467:SpyTag strain for SpyCatcher labelling	Caroline Ajo-Franklin Rice University	1
Plasmid			
pBAD-SpyCatcher-mRFP1	Expression and purification of Spycatcher-mRFP1	Caroline Ajo-Franklin Rice University	1
pDEST14-SpyCatcher-sfGFP	Expression strain and plasmid for SpyCatcher-sfGFP	Mark Howarth University of Oxford	2

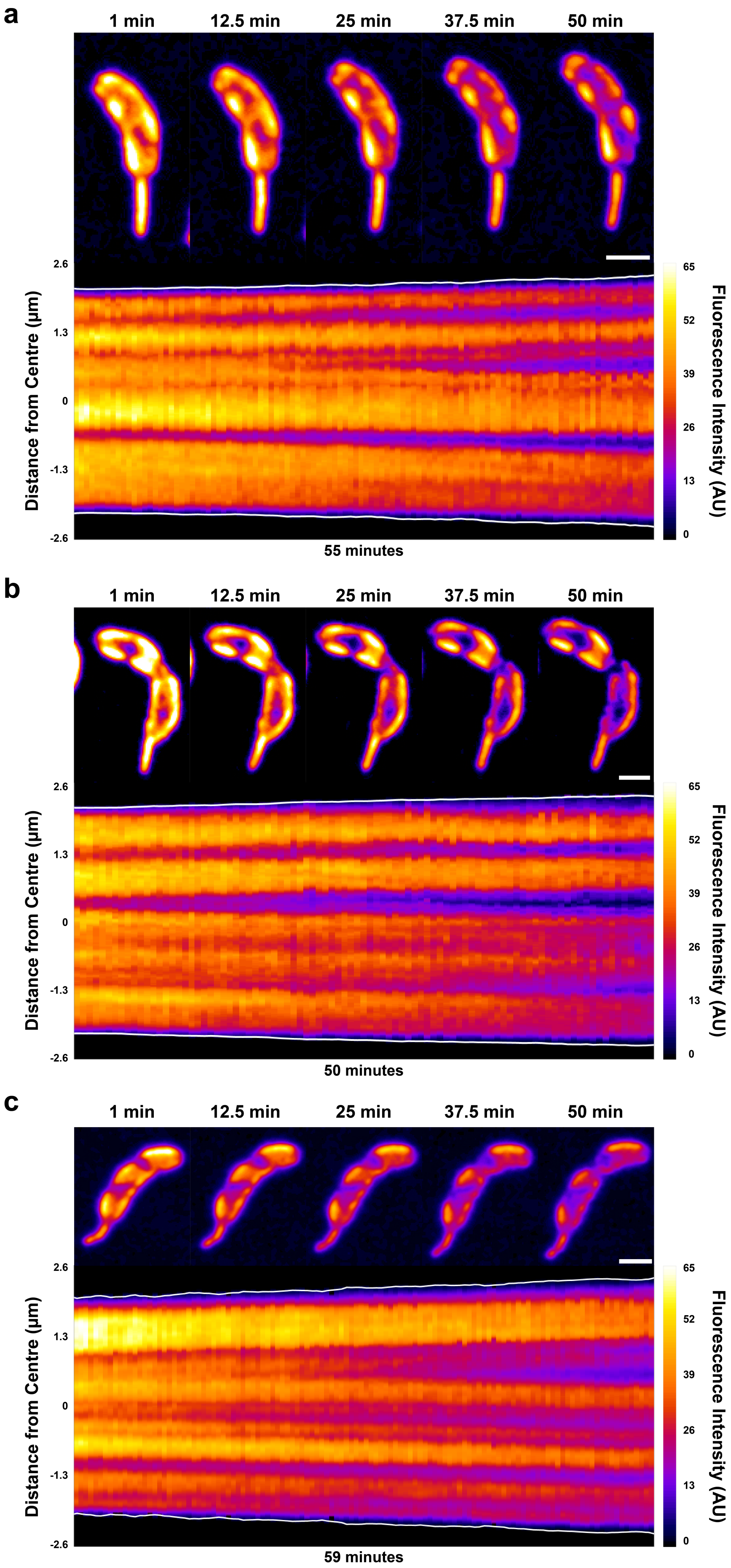
a
1 min
12.5 min
25 min
37.5 min
50 min
Distance from Centre (µm)
2.6
1.3
0
-1.3
-2.6
55 minutes
Fluorescence Intensity (AU)
65
52
39
26
13
0
b
1 min
12.5 min
25 min
37.5 min
50 min
Distance from Centre (µm)
2.6
1.3
0
-1.3
-2.6
50 minutes
Fluorescence Intensity (AU)
65
52
39
26
13
0
c
1 min
12.5 min
25 min
37.5 min
50 min
Distance from Centre (µm)
2.6
1.3
0
-1.3
-2.6
59 minutes
Fluorescence Intensity (AU)
65
52
39
26
13
0

4

Supplementary Fig. 1. Live cell imaging of SC-mRFP1 labelled *C. crescentus* cells.

(**a-c**) *C. crescentus* cells labelled using SC-mRFP1, grown on PYE-agar at 30 °C. Cells were imaged over 1 hour in 30 second intervals. Cell growth and progression of the cell cycle was followed using the kymographs provided for each cell, showing fluorescence along the medial axis. Fluorescent regions remain uniform in size throughout the labelling, consistent with the proposed non-diffusible nature of crystallised RsaA on the cell surface. Time lapse experiments were repeated three times, independently.

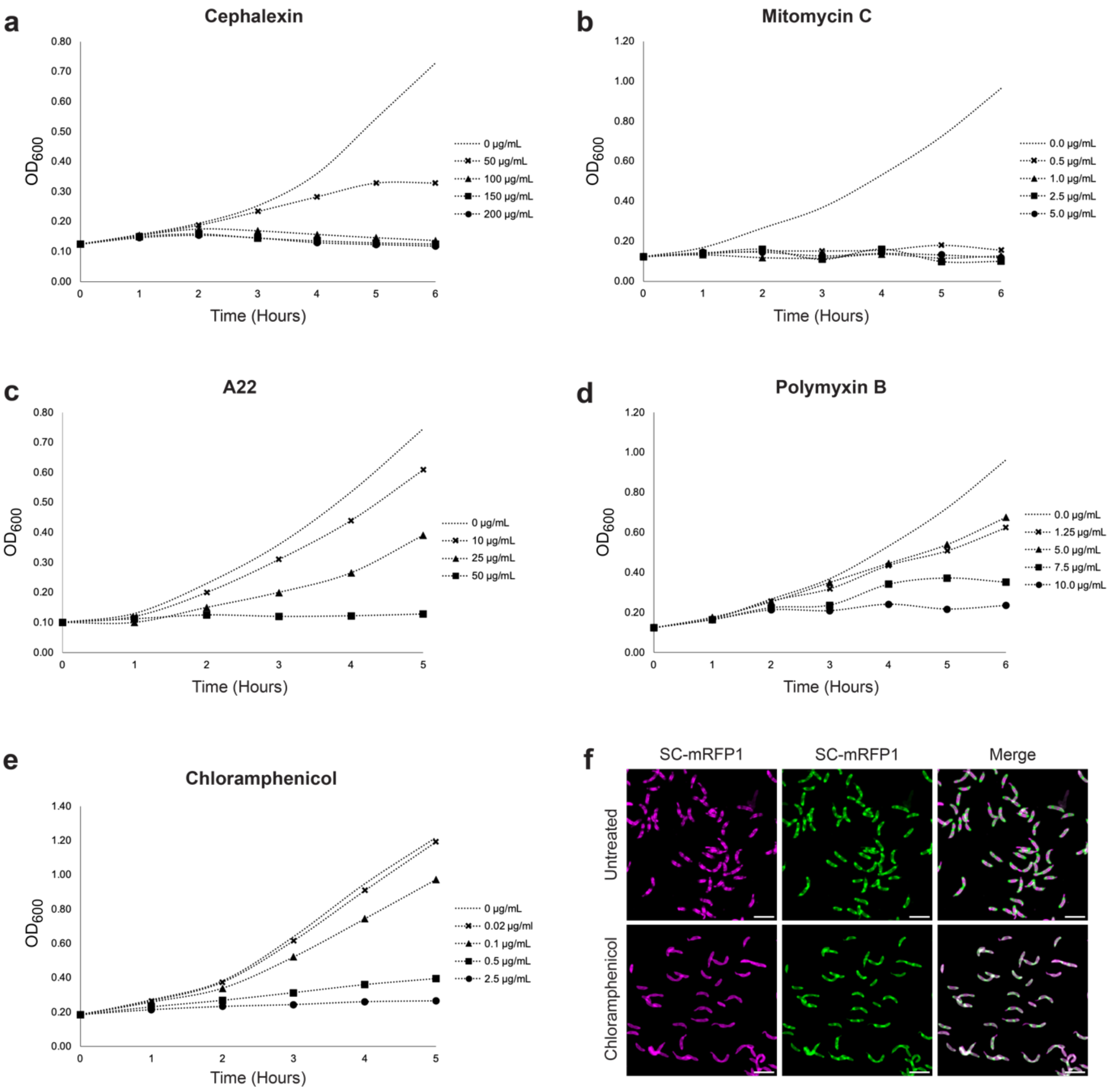

Supplementary Fig. 2. *C. crescentus* growth curves under exposure to various compounds.

C. crescentus rsaA-467-spytag Δ*sapA* grown in the presence of varying concentrations of (**a**) cephalexin, (**b**) mitomycin C (MMC), (**c**) A22, (**d**) polymyxin B, and (**e**) chloramphenicol at to determine optimal (sub-lethal) levels for pulse-chase labelling experiments. All samples were grown in liquid PYE media at 30 °C, with shaking. (**f**) *C. crescentus* cells grown in varying concentrations of chloramphenicol over 5 hours. Pulse-chase labelled in SC-mRFP1 (magenta) and SC-sfGFP (green) in PYE (no treatment) or PYE supplemented with 0.5 µg/mL chloramphenicol. Scale bars = 5 µm. Source data are provided as an accompanying Source Data file.

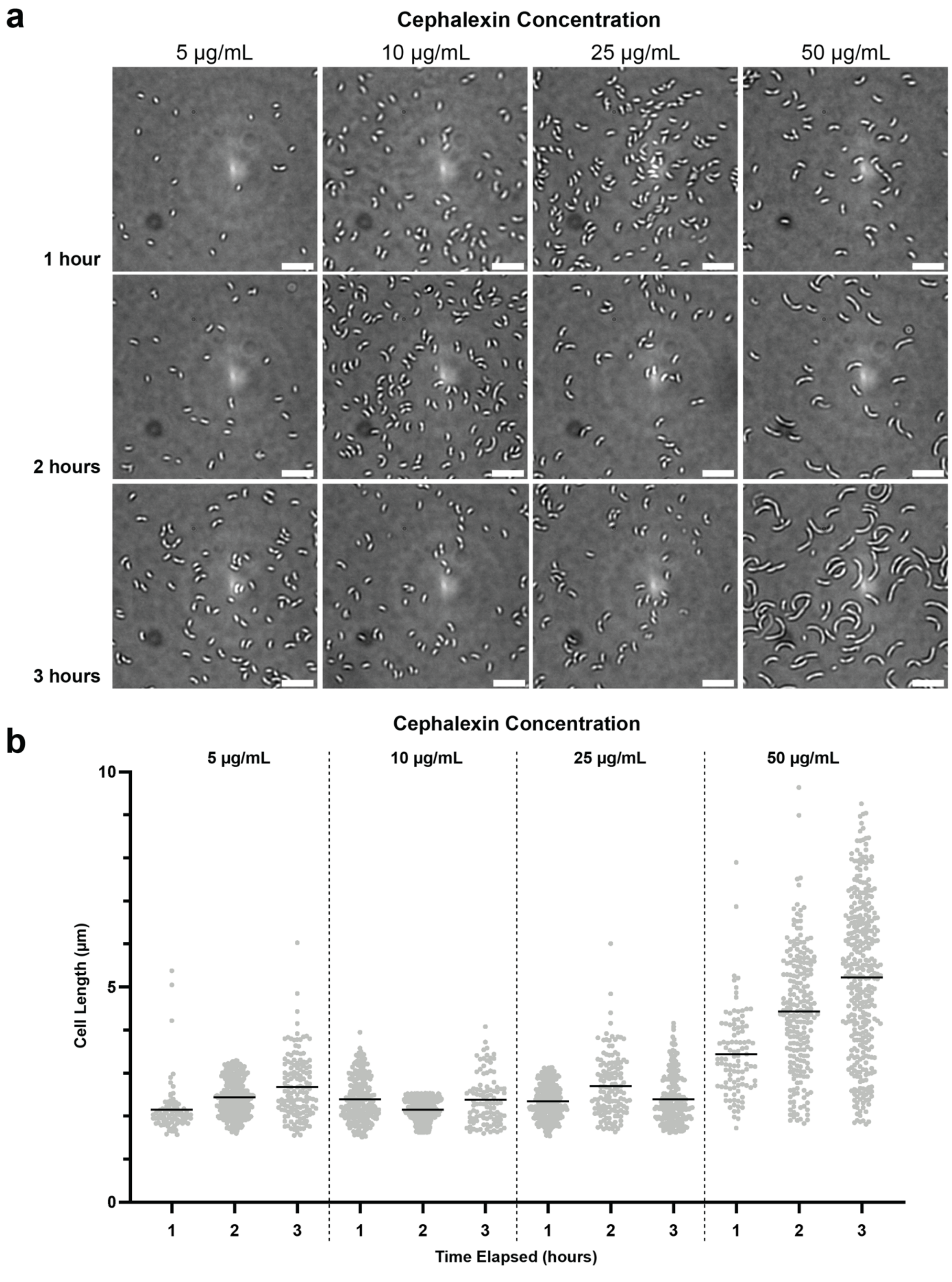

<u>Supplementary Fig. 3. Elongation of *C. crescentus* following incubation with cephalexin.</u>

C. crescentus cells were grown in PYE containing varying concentrations of cephalexin. (**a**) Samples were taken each hour for a total of three hours, spotted onto an agarose pad, and

imaged to observe morphological changes caused by cephalexin exposure. Scale bars = 10 µm. (**b**) Using MicrobeJ, the cell lengths were extracted and quantified, showing that 3 hours of incubation with 50 µg/mL cephalexin results in a drastically increased average cell length (5.22 µm), almost double that of cells grown in 25 µg/mL cephalexin, which led to an average cell length of 2.70 µm after two hours of exposure. $n \geq 85$ cells per group. Data points (represented as ●) show length of individual cells. Source data are provided as an accompanying Source Data file.

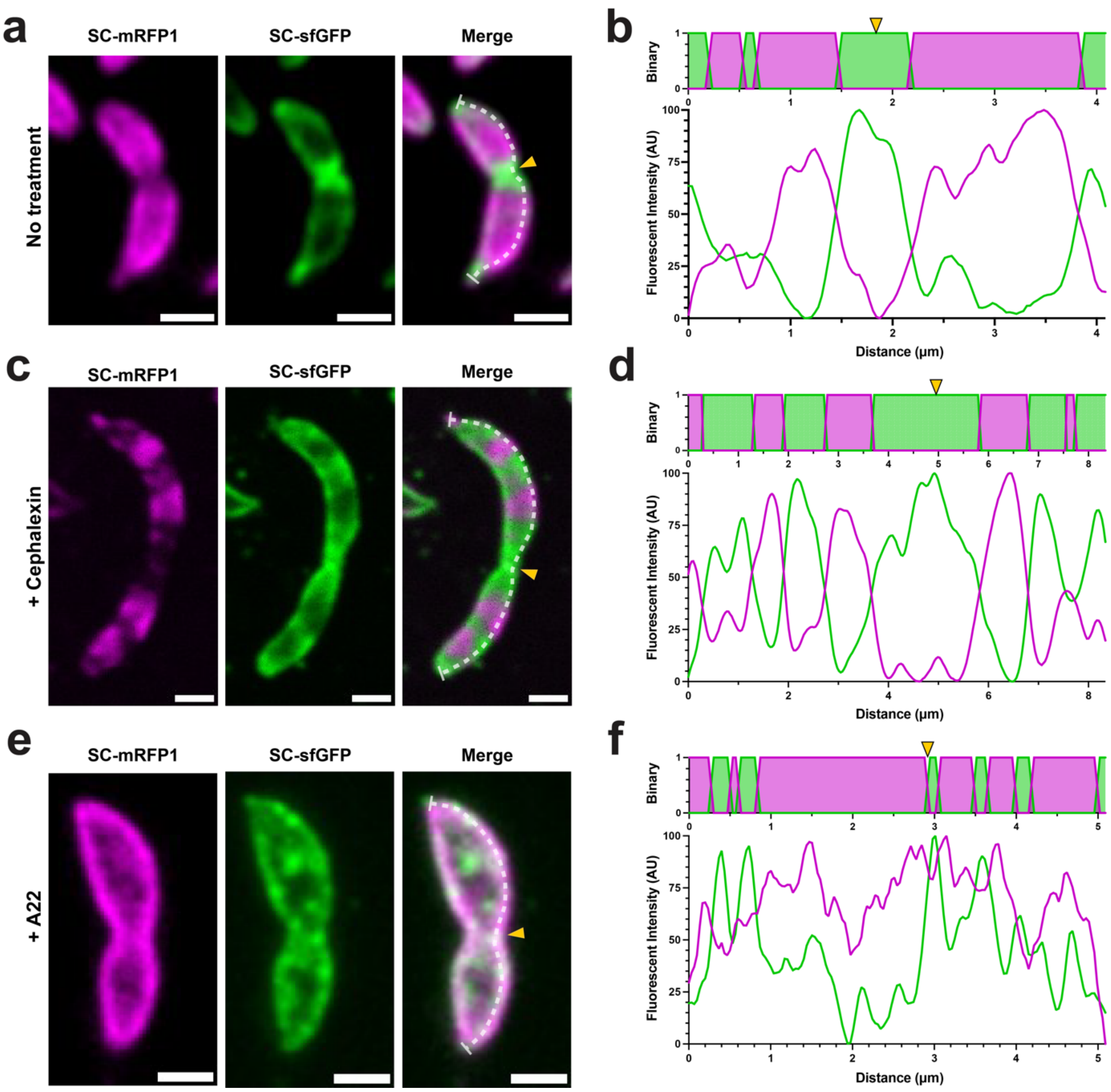

<u>Supplementary Fig. 4. Cephalexin treated *C. crescentus* cells display a similar labelling pattern to untreated cells, markedly different from cells treated with A22.</u>

(**a**) mRFP1 (magenta), sfGFP (green) and merge micrographs of a representative untreated cell. (**b**) A three-pixel line was manually drawn along the right-axes of the cell (white dashed line) and the resulting profiles are displayed to the right of the micrographs. The fluorescence profile (starting from the northmost point of the cell) was normalised and plotted (green and magenta lines) accordioning to the position along the cell axis. Above the normalised fluorescence data, a binary projection of the two channels is presented to show the dominant signal along the cell. The mid-cell, as determined by the presence of invagination, is indicated by a yellow arrow on

the merged image and the binary cell profile. (**c**-**d**) Corresponding mRFP1, sfGFP and merge micrographs of a representative cephalexin-treated cell (50 μg/mL) along with the extracted profiles, presented as described above. (**e**-**f**) Corresponding mRFP1, sfGFP and merge micrographs of a representative A22-treated cell (3 μg/mL) along with the extracted profiles, presented as described above. Scale bars = 1 μm.

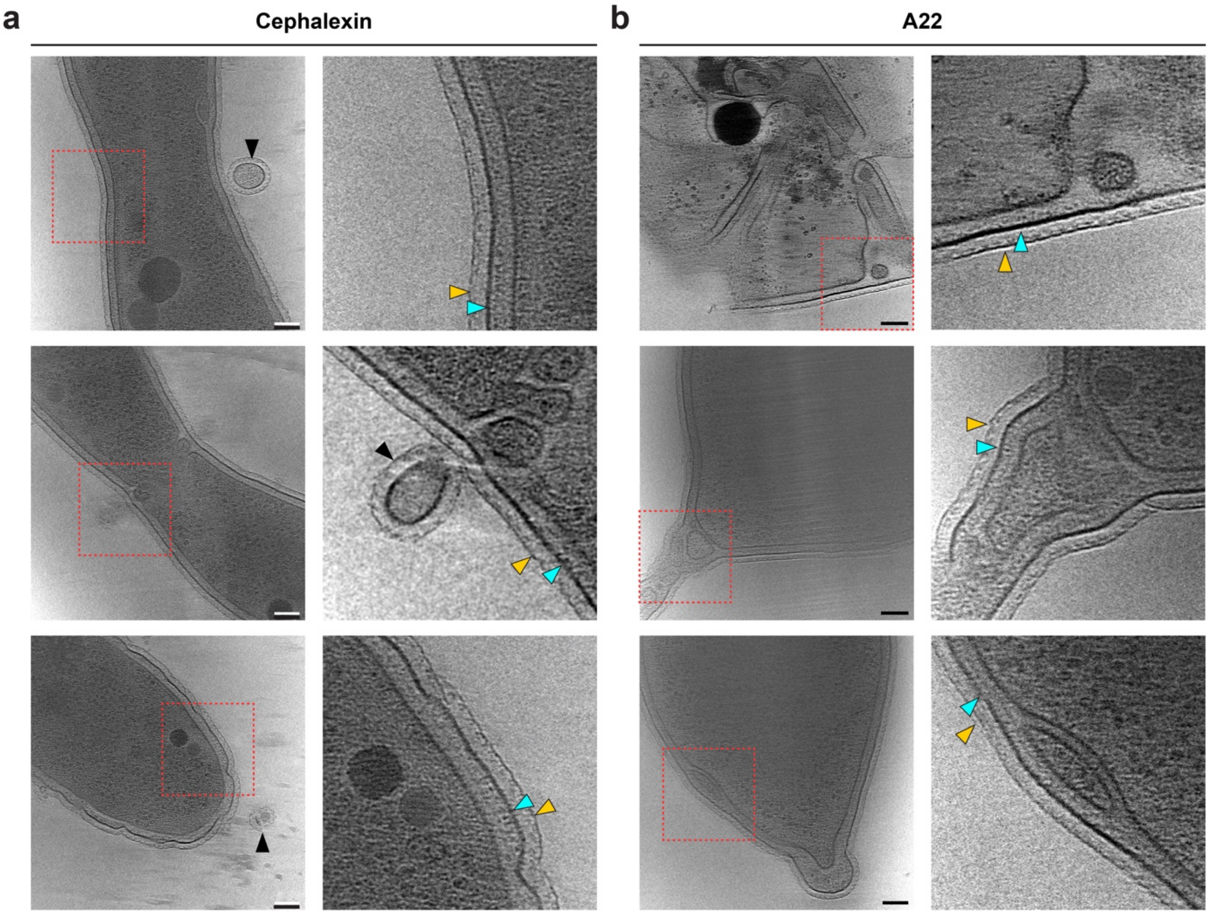

Supplementary Fig. 5. Cryo-ET gallery of cephalexin and A22 treated *C. crescentus* cells.

Slices through reconstructed tomograms of (**a**) cephalexin (50 µg/mL) and (**b**) A22 (3 µg/mL) treated cells. The left panel in each case shows a view of the cell body (scale bars = 100 nm), while the right panel shows a zoomed-in view of the regions highlighted by the red box. Z-slice has been adjusted in the zoomed views to highlight the cell envelope.

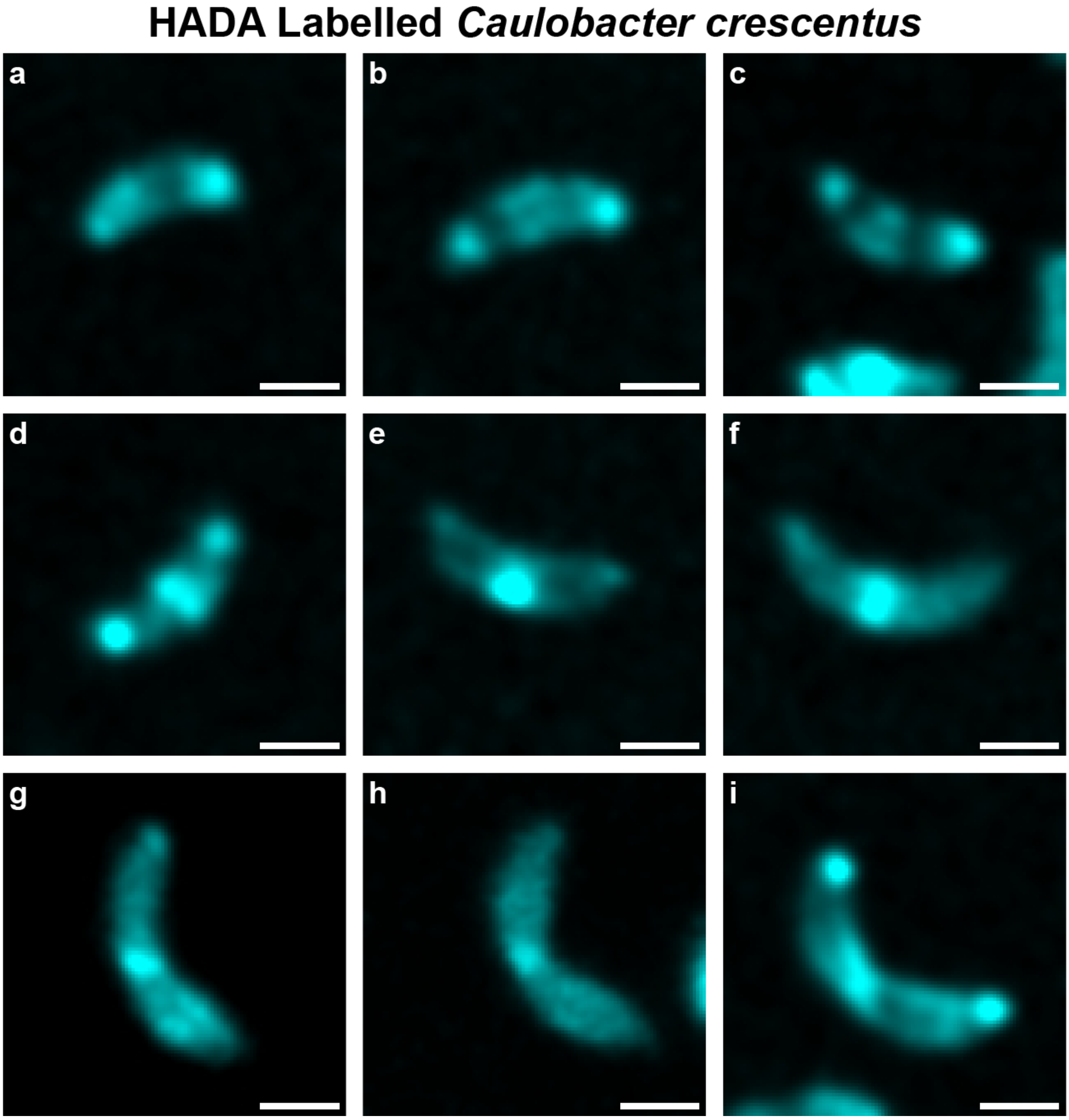

Supplementary Fig. 6. Gallery of HADA labelled *C. crescentus* cells.

(**a-i**) Micrographs showing HADA-labelled *C. crescentus* cells arranged by ascending cell length. Scale bars = 1 µm.

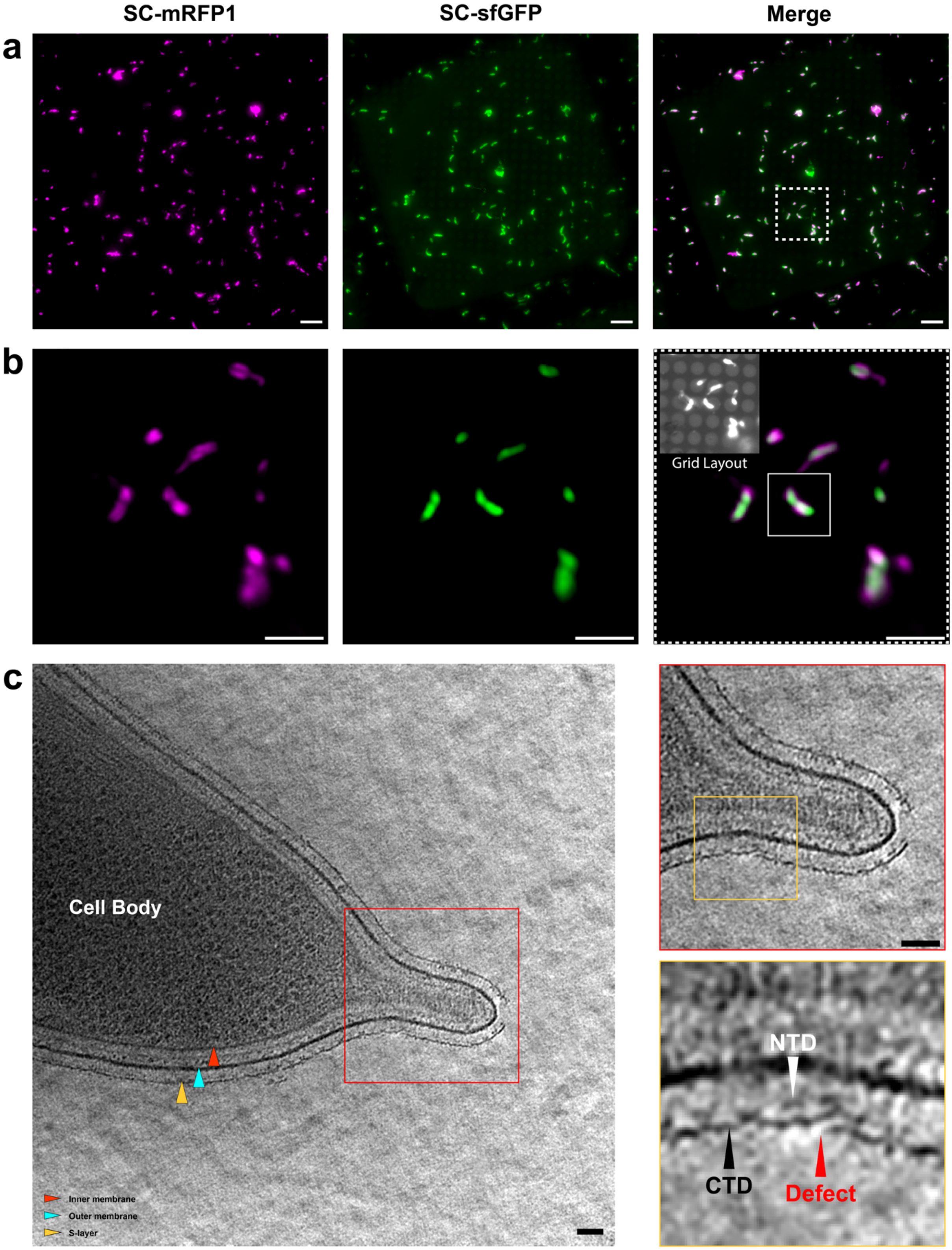

Supplementary Fig. 7. Cryo-CLEM experiment to confirm that RsaA inserts at disruptions in the cellular S-layer.

(**a**) Maximum Z-projection of a Z-stack through a cryo-EM grid containing vitrified, dual-labelled *C. crescentus* cells. Scale bars = 10 µm. Regions of old S-layer are highlighted in magenta (SC-mRFP1), new S-layer in green (SC-sfGFP), and a merge of both channels is provided in the last panel. (**b**) A zoomed view of the subsection of the micrograph showing the region used for cryo-ET data collection, highlighted in the merged panel of a) (white dashed border), channels are arranged as in a). Scale bars = 5 µm. The inset in the merged channel micrograph shows a re-contrasted image where the layout of the EM grid, and the cell selected for cryo-ET collection is highlighted by a white box. (**c**) Slice through a tomogram of the dual-labelled *C. crescentus* cell highlighted in b). Components of the cell envelope are labelled using coloured arrows (legend in the bottom left of the panel). A zoomed view of the region highlighted by the red box in panel c), showing a short stalk. Discontinuity in the S-layer can be seen at the stalk tip (likely due to the presence of the holdfast-polysaccharide[110]) and at the base of the stalk. Scale bars = 50 nm. A further closeup of the latter is given in the bottom right panel, showing overlapping regions of the S-layer (labelled as "Defect" using a red arrow). The N-terminal and C-terminal domains (NTD and CTD) of RsaA in the assembled S-layer are marked by white and black arrows, respectively.

SUPPLEMENTARY REFERENCES

1 Charrier, M. *et al.* Engineering the S-Layer of Caulobacter crescentus as a Foundation for Stable, High-Density, 2D Living Materials. *ACS Synth Biol* **8**, 181-190 (2019). https://doi.org:10.1021/acssynbio.8b00448

2 Keeble, A. H. *et al.* Approaching infinite affinity through engineering of peptide–protein interaction. *Proceedings of the National Academy of Sciences* **116**, 26523-26533 (2019).
