# Supplementary material for: Cell cycle dependent coordination of surface layer biogenesis in Caulobacter crescentus
Source: Nat Commun. 2024 Apr 18;15:3355. doi: 10.1038/s41467-024-47529-5 (PMC11026435; doi:10.1038/s41467-024-47529-5)
Supplement: Supplementary file 3 — Description of Additional Supplementary Files [file 41467_2024_47529_MOESM3_ESM.pdf]

Description of Additional Supplementary Files:

Supplementary Movie 1: Tomogram of cephalexin-treated C. crescentus cell.

Supplementary Movie 2: Tomogram of A22- treated C. crescentus cell.

Supplementary Movie 3: Tomogram of A22- treated C. crescentus cell with envelope folding.
