# Supplementary material for: Cell cycle dependent coordination of surface layer biogenesis in Caulobacter crescentus
Source: Nat Commun. 2024 Apr 18;15:3355. doi: 10.1038/s41467-024-47529-5 (PMC11026435; doi:10.1038/s41467-024-47529-5)
Supplement: Supplementary file 7 — Reporting Summary [file 41467_2024_47529_MOESM7_ESM.pdf]

nature portfolio

Corresponding author(s): Tanmay Bharat

Last updated by author(s): Mar 8, 2024

Reporting Summary

Nature Portfolio wishes to improve the reproducibility of the work that we publish. This form provides structure for consistency and transparency in reporting. For further information on Nature Portfolio policies, see our Editorial Policies and the Editorial Policy Checklist.

Statistics

For all statistical analyses, confirm that the following items are present in the figure legend, table legend, main text, or Methods section.

n/a	Confirmed	
☐	☒	The exact sample size (*n*) for each experimental group/condition, given as a discrete number and unit of measurement
☐	☒	A statement on whether measurements were taken from distinct samples or whether the same sample was measured repeatedly
☐	☒	The statistical test(s) used AND whether they are one- or two-sided<br>*Only common tests should be described solely by name; describe more complex techniques in the Methods section.*
☐	☒	A description of all covariates tested
☐	☒	A description of any assumptions or corrections, such as tests of normality and adjustment for multiple comparisons
☐	☒	A full description of the statistical parameters including central tendency (e.g. means) or other basic estimates (e.g. regression coefficient) AND variation (e.g. standard deviation) or associated estimates of uncertainty (e.g. confidence intervals)
☐	☒	For null hypothesis testing, the test statistic (e.g. *F*, *t*, *r*) with confidence intervals, effect sizes, degrees of freedom and *P* value noted<br>*Give P values as exact values whenever suitable.*
☒	☐	For Bayesian analysis, information on the choice of priors and Markov chain Monte Carlo settings
☒	☐	For hierarchical and complex designs, identification of the appropriate level for tests and full reporting of outcomes
☐	☒	Estimates of effect sizes (e.g. Cohen's *d*, Pearson's *r*), indicating how they were calculated

Our web collection on statistics for biologists contains articles on many of the points above.

Software and code

Policy information about availability of computer code

Data collection	Light microscopy: Cells were imaged using an Olympus SoRa spinning disc confocal microscope equipped with Olympus CellSens software, an Olympus IX-83 inverted frame, a Yokogawa SoRa super-resolution spinning disc module, and Prime BSI camera. Slides were kept at room temperature and imaged using the 60x (1.5 NA) lens, with excitation at 488 nm (sfGFP) and 561 nm (mRFP1) using solid state lasers at 200 ms exposure. Z-stacks were taken at 0.26 µm intervals and an Olympus Super Resolution filter applied to the entire stack, followed by deconvolution using a maximum likelihood algorithm (5 iterations). For HADA-labelling experiments, cells were imaged using an Olympus Fluoview FV1200 equipped with equipped with GaAsP detectors. Images were acquired using a 100 x (1.4 NA) lens with excitation via solid state 405 nm, 559 nm, and argon 488 nm lasers, with scanning at 1024 x 1024 pixels. A Kalman filter (2 iterations) was applied for all image collections. Single slices through the middle of the cells were taken, without Z-stacks. ROIs were selected randomly following mapping of the mounted slide. Regions of cell crowding, uneven agarose, or devoid of cells were excluded.<br>Cryoelectron tomography: Tomographic was collected using SerialEM 3 software. Collection was carried out using the Quantum energy filter (slit width 20 eV) and the K2 or K3 direct electron detector running in counting mode. Tilt series with a defocus range of -5 to -8 µm were collected between ± 65° in a bidirectional (only for Fig. 8a-c) or ±60º dose symmetric scheme with a 1° tilt increment. A total dose of 150 e-/A˚2 (only Fig. 8a-c) or 73 e-/A˚2 was applied over the entire series.

Data analysis	Light microscopy: Pearson's Correlation Coefficient (PCC) was calculated using Coloc2 plugin for ImageJ/FIJI. The MicrobeJ plugin for ImageJ/FIJI was used to generate data for cell demographs, kymographs, and fluorescent profiles of individual cells. Graphs, with the exception of cell profile plots and demographs, were generated in and statistical analyses were carried out using GraphPad Prism 9.5.1. Where appropriate, the number of measurements performed are reported in the relevant figure legends.<br>Cryoelectron tomography: Cryo-ET data analysis was performed in IMOD and tomographic reconstruction was carried out using the SIRT algorithm implemented within Tomo3D. Datasets were motion corrected and dose weighted with MotionCor2 implemented in Relion 3.0. Contrast transfer functions (CTFs) of the resulting motion corrected micrographs were estimated using CTFFIND4.

For manuscripts utilizing custom algorithms or software that are central to the research but not yet described in published literature, software must be made available to editors and reviewers. We strongly encourage code deposition in a community repository (e.g. GitHub). See the Nature Portfolio guidelines for submitting code & software for further information.

Data

Policy information about availability of data

All manuscripts must include a data availability statement. This statement should provide the following information, where applicable:

- Accession codes, unique identifiers, or web links for publicly available datasets
- A description of any restrictions on data availability
- For clinical datasets or third party data, please ensure that the statement adheres to our policy

The source data underlying Figures with plotted graphs is available with this manuscript as a Source Data file. All other data, including cryo-ET and light microscopy images, are available from the authors upon request.

Research involving human participants, their data, or biological material

Policy information about studies with human participants or human data. See also policy information about sex, gender (identity/presentation), and sexual orientation and race, ethnicity and racism.

Reporting on sex and gender	Our studies involve no human participants, their data, or biological material.
Reporting on race, ethnicity, or other socially relevant groupings	Our studies involve no human participants, their data, or biological material.
Population characteristics	Our studies involve no human participants, their data, or biological material.
Recruitment	Our studies involve no human participants, their data, or biological material.
Ethics oversight	Our studies involve no human participants, their data, or biological material.

Note that full information on the approval of the study protocol must also be provided in the manuscript.

Field-specific reporting

Please select the one below that is the best fit for your research. If you are not sure, read the appropriate sections before making your selection.

☒ Life sciences ☐ Behavioural & social sciences ☐ Ecological, evolutionary & environmental sciences

For a reference copy of the document with all sections, see nature.com/documents/nr-reporting-summary-flat.pdf

Life sciences study design

All studies must disclose on these points even when the disclosure is negative.

Sample size	Sample sizes were chosen based on standards within the field for biological replicates. Each reported experiment is representative of at least three biological replicates. Sample sizes were deemed satisfactory following statistical analysis and confirmation or absence of statistical difference between conditions.
Data exclusions	No data was excluded from the analyses.
Replication	All experiments consisted of at least three successful repeats, resulting in comparable results each time. Micrographs presented together in the study are representative of one of the repeated experiments.
Randomization	Light microscopy image collection was randomized through random selection of regions of interest (ROI) across slide-mounted samples, to eliminate personal bias. Cells in each ROI were included in the analysis, with the exceptions noted in the manuscript methods (i.e. obscured cells or cells cut off by the field of view).
Blinding	Blinding was found to be unnecessary for this study. Variables were accounted for and the data analysis methods used (e.g. PCC, statistical analyses) eliminate significant impact subjectivity may have on the interpretation of results. Negative controls (i.e., untreated cells) were also used for relevant experiments.

Reporting for specific materials, systems and methods

We require information from authors about some types of materials, experimental systems and methods used in many studies. Here, indicate whether each material, system or method listed is relevant to your study. If you are not sure if a list item applies to your research, read the appropriate section before selecting a response.

Materials & experimental systems

n/a	Involved in the study
☒	☐ Antibodies
☒	☐ Eukaryotic cell lines
☒	☐ Palaeontology and archaeology
☒	☐ Animals and other organisms
☒	☐ Clinical data
☒	☐ Dual use research of concern
☒	☐ Plants

Methods

n/a	Involved in the study
☒	☐ ChIP-seq
☒	☐ Flow cytometry
☒	☐ MRI-based neuroimaging

Plants

Seed stocks

Report on the source of all seed stocks or other plant material used. If applicable, state the seed stock centre and catalogue number. If plant specimens were collected from the field, describe the collection location, date and sampling procedures.

Novel plant genotypes

Describe the methods by which all novel plant genotypes were produced. This includes those generated by transgenic approaches, gene editing, chemical/radiation-based mutagenesis and hybridization. For transgenic lines, describe the transformation method, the number of independent lines analyzed and the generation upon which experiments were performed. For gene-edited lines, describe the editor used, the endogenous sequence targeted for editing, the targeting guide RNA sequence (if applicable) and how the editor was applied.

Authentication

Describe any authentication procedures for each seed stock used or novel genotype generated. Describe any experiments used to assess the effect of a mutation and, where applicable, how potential secondary effects (e.g. second site T-DNA insertions, mosiacism, off-target gene editing) were examined.
